# Metabolic pathways regulated by TAp73 in response to oxidative stress

**DOI:** 10.18632/oncotarget.8935

**Published:** 2016-04-22

**Authors:** Massimiliano Agostini, Margherita Annicchiarico-Petruzzelli, Gerry Melino, Alessandro Rufini

**Affiliations:** ^1^ Medical Research Council, Toxicology Unit, Leicester University, Leicester, UK; ^2^ Department of Experimental Medicine and Surgery, University of Rome “Tor Vergata”, Rome, Italy; ^3^ Biochemistry Laboratory IDI-IRCC, Department of Experimental Medicine and Surgery, University of Rome “Tor Vergata”, Rome, Italy; ^4^ Department of Cancer Studies, CRUK Leicester Cancer Centre, University of Leicester, Leicester, UK

**Keywords:** p73, p53 family, oxidative stress, metabolism, ROS

## Abstract

Reactive oxygen species are involved in both physiological and pathological processes including neurodegeneration and cancer. Therefore, cells have developed scavenging mechanisms to maintain redox homeostasis under control. Tumor suppressor genes play a critical role in the regulation of antioxidant genes. Here, we investigated whether the tumor suppressor gene TAp73 is involved in the regulation of metabolic adaptations triggered in response to oxidative stress. H_2_O_2_ treatment resulted in numerous biochemical changes in both control and TAp73 knockout (TAp73−/−) mouse embryonic fibroblasts, however the extent of these changes was more pronounced in TAp73−/− cells when compared to control cells. In particular, loss of TAp73 led to alterations in glucose, nucleotide and amino acid metabolism. In addition, H_2_O_2_ treatment resulted in increased pentose phosphate pathway (PPP) activity in null mouse embryonic fibroblasts. Overall, our results suggest that in the absence of TAp73, H_2_O_2_ treatment results in an enhanced oxidative environment, and at the same time in an increased pro-anabolic phenotype. In conclusion, the metabolic profile observed reinforces the role of TAp73 as tumor suppressor and indicates that TAp73 exerts this function, at least partially, by regulation of cellular metabolism.

## INTRODUCTION

The maintenance of redox homeostasis is a crucial task for the cell, as different levels of reactive oxygen species can induce different biological responses, often associated with pathologies such as cancer and neurodegeneration [1–10]. High levels of ROS are detrimental, whereas at low levels, ROS sustains differentiation and proliferation, therefore acting as signaling molecules [11–21]. Indeed, cells can produce hydrogen peroxide (H_2_O_2_) in order to modulate biological processes as diverse as proliferation, differentiation and migration [22–26]. On the other hand, excessive production of ROS leads to the deleterious oxidative damage [27–31]. Hence, cells have developed numerous ROS scavenging mechanisms [32–36], most notably GSH [37, 38], catalase and superoxide dismutase and, of note, most of them are regulated by different tumor suppressor genes to safeguard cellular redox homeostasis counteracting excessive ROS production [39–41]. Among the tumor suppressor genes, the p53-family (p53, p63 and p73 proteins) [42–50] has a key role in controlling antioxidant gene expression [51–54]. Indeed, p53 regulates the expression of numerous antioxidant genes, including, sestrins, *TIGAR* and glutaminase-2 (*GLS2*) [55–58], thus contributing to ROS homeostasis.

Recent studies have also demonstrated an essential role for p73 and p63 in regulation of oxidative metabolism. In fact, deletion of the long TAp73 isoform of p73 increases ROS production and oxidative stress by affecting electron flux during mitochondrial oxidative phosphorylation and flux through the oxidative arm of the PPP [59–61]. Similarly, p63 contributes to the maintenance of a balanced redox state in keratinocytes and lung cancer cells through the regulation of *GLS2*, cytoglobin, hexokinase-II and *REDD1* [62–65].

The aim of this study was to identify the differences in global biochemical responses to oxidative stress between wild-type and TAp73 knock-out (TAp73−/−) mouse embryonic fibroblasts (MEFs), with the held hypothesis that TAp73 controls oxidative metabolism and response to oxidative stress. H_2_O_2_ treatment resulted in numerous biochemical changes in both WT and TAp73−/− cells, but the number and extent of these changes was more robust in TAp73−/− cells as compared to WT control. Overall, it appears that in the absence of TAp73, H_2_O_2_ treatment results in an enhanced oxidative environment, possibly promoted by an increased nucleotide catabolism, concomitant to a decreased apoptotic biochemical profile as compared to TAp73-proficient cells.

## RESULTS

### H_2_O_2_ induced-oxidative stress and glutathione recycling is potentially greater in TAp73−/− versus WT MEFs

In order to explore the metabolic role of TAp73 in oxidative stress, MEF derived from TAp73−/− and control mice were treated with H_2_O_2_ and then subjected GC-MS and LC-MS-MS platforms for metabolomics studies as previously described [66]. The total numbers of significantly or nearly-significantly altered biochemicals are reported in Table S1.

**Figure 1:**
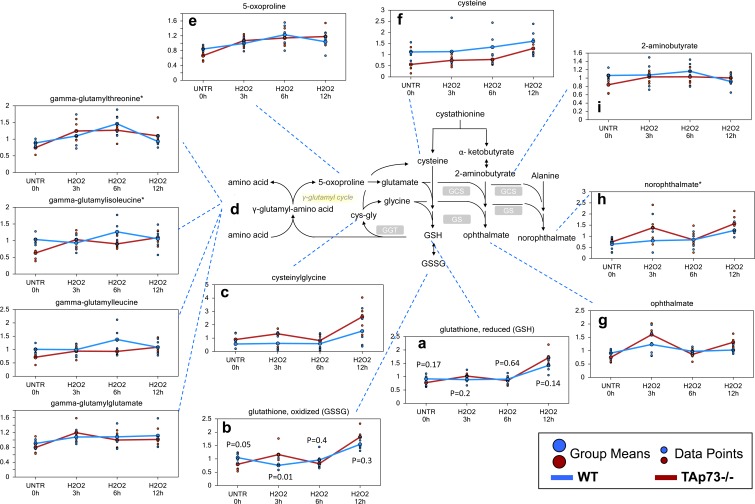
Glutathione recycling is potentially greater in TAp73−/− *versus* WT MEF GSH is a key antioxidant molecule within the cell. The availability of the amino acid precursor, cysteine, and the activity of the rate-limiting enzyme, glutamate cysteine ligase, are the key factors in GSH synthesis. **a.**-**i.** Levels of the indicated metabolites were evaluated as described in material and methods. Anova contrasts *t*-tests were used to identify biochemicals that differed significantly between experimental groups (*n* = 5 for each time point). P-values for reduced and oxidized glutathione are also reported for each time point.

The tripeptide glutathione (gamma-glutamyl-cysteinylglycine) functions as one of the major antioxidants in cells [67]. Both reduced and oxidized glutathione (GSH and GSSG) levels were increased following the H_2_O_2_ treatment time course in the WT and TAp73−/− cells, but these increases were greater in TAp73−/− cells (Figure 1a and 1b). In addition, biochemicals associated with increased glutathione recycling (cysteinylglycine, gamma-glutamyl-amino acids, and 5-oxoproline) were also more elevated in the TAp73−/− cells, suggesting an increased rate of glutathione turnover occurring in the TAp73−/− cells over the course of H_2_O_2_ treatments (Figure 1c-1e). Cysteine, which is the rate-limiting precursor to glutathione [68], showed increased levels in both WT and TAp73−/− cells during the H_2_O_2_ treatment and this increase was more pronounced and reached statistical significance in TAp73−/− cells. However, the absolute levels of cysteine remained consistently lower in the TAp73−/− cells, suggesting reduced cysteine precursor for glutathione biosynthesis (Figure 1f). The increased glutathione levels in both WT and TAp73−/− MEFs during the time course suggest that cysteine biosynthesis is enhanced by H_2_O_2_ in order to fuel the supply of glutathione. It should be noted that, in untreated cells (UNTR) the levels of cysteine were significantly lower in TAp73−/− as compared to WT, and remained such throughout the H_2_O_2_ time course. In keeping with the reduced cysteine levels in TAp73−/− cells, we identified increased levels of the tripeptides opthalmate (gamma-glutamyl-alpha-aminobutyrylglycine) (Figure 1g) and norophthalmate (gamma-glutamyl-alanylglycine) (Figure 1h) in knockout cells as compared to WT controls following H_2_O_2_ treatment. 2-aminobutyrate and alanine replace cysteine during the synthesis of ophthalmate and norophthalmate respectively (Figure 1i). Thus, the increase in ophthalmate and norophthalmate could suggest either adaptation to limiting cysteine levels or to augmented glutathione synthetase (GCS) activity, triggered by oxidative environment. Increased levels of the oxidative by-product of sterols, such as oxysterols, 7-ketocholesterol and 7-beta-hydroxycholesterol further support an increased oxidative environment in the TAp73−/− cells as compared to WT cells (Table S1).

### Methionine metabolism is enhanced following H_2_O_2_ treatment predominately in TAp73−/− cells

#### Cysteine biosynthesis

**Figure 2:**
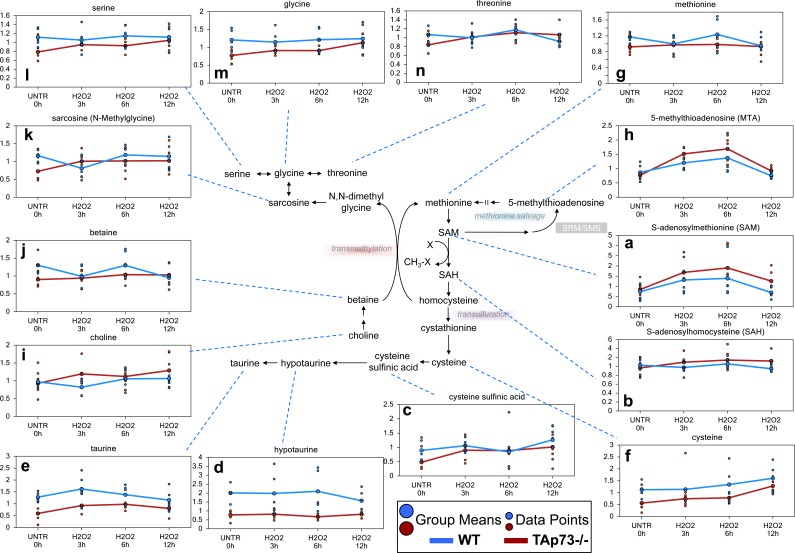
Loss of TAp73 enhances methionine metabolism following H_2_O_2_ treatment Methionine is the initiating amino acid in the synthesis of eukaryotic proteins. Methionine metabolism begins with its activation to SAM by methionine adenosyltransferase. **a.**-**n.** Levels of the indicated metabolites were evaluated as described in material and methods. Anova contrasts *t*-tests were used to identify biochemicals that differed significantly between experimental groups (*n* = 5 for each time point).

As previously stated, cysteine levels were elevated in both WT and TAp73−/− cells over the H_2_O_2_ treatment time course, but these increases were more robust in TAp73−/− cells. The major source for cysteine biosynthesis is through methionine metabolism [69]. H_2_O_2_ treatment induced significant increases in the methionine metabolite, S-adenosylmethionine (SAM), in both the WT and TAp73−/− cells, with TAp73−/− cells having more robust changes (Figure 2a). In addition, S-adenosylhomosysteine (SAH), which is formed when SAM participates in methylation events, demonstrated a trend of increasing levels in TAp73−/− over the H_2_O_2_ time course, but this increase did not reach significance, while SAH was unchanged over time in WT (Figure 2b). One possible explanation for why SAH showed non-significant increases in TAp73−/− and was unchanged in WT could be due to increased metabolism to homocysteine and subsequently to cystathionine to fuel cysteine biosynthesis. The previously described increase in cysteine supports this possibility. Not only can cysteine be metabolized to glutathione, but it can also be oxidized to cysteine sulfinic acid, which can be further metabolized to hypotaurine and taurine. This metabolic route further depletes the cells of cysteine for glutathione synthesis. While cysteine sulfinic acid was increased in WT cells depending upon the H_2_O_2_ time point investigated, this increase never reached significance, and neither hypotaurine nor taurine were significantly changed in WT (Figure 2c-2e). In contrast, cysteine sulfinic acid was significantly elevated in the TAp73−/− cells following H_2_O_2_ treatment as compared to UNTR cells (Figure 2c), and although hypotaurine was unchanged, taurine was also significantly elevated in the TAp73−/− cells over the H_2_O_2_ time course (Figure 2d and 2e). The lack of change in hypotaurine in TAp73−/− cells may reasonably result from subsequent metabolism to taurine. Thus, the already lower pool of cysteine in TAp73−/− cells (Figure 2f) appears to be further decreased by conversion to cysteine sulfinic acid at a higher rate than that seen in WT and may have adverse effects on the synthesis of glutathione and thus compromise redox homeostasis in the TAp73−/− cells.

#### Methionine salvage and transmethylation

Although there appeared to be an increase in methionine metabolism in both the WT and TAp73−/− cells, methionine levels were only significantly lower at the 12 hour H2O2 time point in the WT cells and were unchanged throughout the H_2_O_2_ treatments in the TAp73−/− cells (Figure 2g), which may suggest increased methionine salvage at the earlier time points in WT and TAp73−/− cells. Increased methionine salvage was supported by the significant increase in 5-methylthioadenosine (MTA) in both WT and TAp73−/− cells following 3 and 6 hours H_2_O_2_ treatment, and this treatment-induced increase was greater in the TAp73−/− cells as compared to WT cells (Figure 2h). In addition to increased methionine salvage, it is possible that increased transmethylation following H_2_O_2_ treatment also contributed to the lack of change in methionine levels in the TAp73−/− cells. Elevated transmethylation in H_2_O_2_-treated TAp73−/− cells was supported by changes in choline, betaine, and sarcosine, glycine, serine and threonine in the TAp73−/− cells (Figure 2i-2n). Briefly, choline can be oxidized to betaine, and betaine can be further metabolized to N,N-dimethylglycine by functioning as a methyl source for the transmethylation of homocysteine back to methionine. Although N,N-dimethylglycine was below the level of detection in TAp73−/− cells, its metabolite sarcosine was increased in the TAp73−/− cells throughout the H_2_O_2_ treatment time course, further supporting increased transmethylation. Sarcosine is rapidly degraded to the amino acids glycine, and glycine can be further metabolized to either threonine or serine. Thus, the increases observed in these amino acids support increased transmethylation activity. It is possible that the increase in sarcosine in the TAp73−/− cells is a *consequence* of increased glycine, which can be methylated to sarcosine, and thus would suggest increased amino acid uptake in the TAp73−/− cells is responsible for the increased sarcosine. Although increased amino acid uptake is possible, due to the additional changes observed for methionine metabolism, increased transmethylation remains a likely explanation for the observed changes in methionine-associated biochemicals.

### Effects on nucleotide metabolism by H_2_O_2_ treatment of TAp73−/− are more profound compared to WT

#### Purine associated metabolites

**Figure 3:**
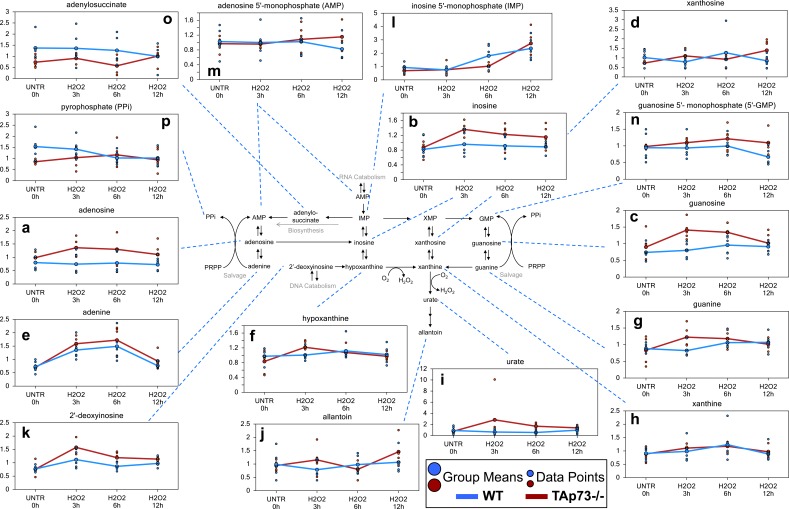
Purine associated metabolites **a.**-**p.** Levels of the indicated metabolites were evaluated as described in material and methods. Anova contrasts *t*-tests were used to identify biochemicals that differed significantly between experimental groups (*n* = 5 for each time point).

Interestingly, only the TAp73−/− cells demonstrated significant or trending increases in the purine nucleosides (adenosine, inosine, and guanosine, and xanthosine) (Figure 3a-3d) and nucleobases (adenine, hypoxanthine, and guanine) following 3 hours H_2_O_2_ treatment (Figure 3e-3g). Both hypoxanthine and guanine can be metabolized to xanthine (Figure 3h), which is subsequently metabolized to urate (Figure 3i) and allantoin (Figure 3j). Xanthine increase initiated at 3 hours of treatment and reached significance at 6 hours in the TAp73−/− cells. We also identified a large, but not significant, increase in urate in TAp73−/− cells following H_2_O_2_ treatment, and this probably fuelled the significant increase in allantoin at the 12 hours H_2_O_2_ treatment time point. The increases in urate and allantoin would suggest that purine catabolism further increased H_2_O_2_ levels in the TAp73−/− cells potentiating the effects of H_2_O_2_ treatment in these cells. In contrast, in WT cells, only xanthine showed an increase, which only trended towards significance. Probably xanthine increase was fuelled by direct conversion of its precursor 2′-deoxyinosine (Figure 3k), which was significantly increased at 3 hours in both WT and TAp73−/− cells. Once again this increase was not only larger in the TAp73−/− cells, but 2′deoxyinosine was also significantly elevated in TAp73−/− cells throughout the entire H_2_O_2_ time course. These changes suggest increased purine catabolism following H_2_O_2_ treatment is more severe in TAp73−/− cells.

In addition to increases in purine catabolites, there was also an increase in the nucleotides, inosine 5′monophosphate (IMP), adenosine 5′-monophosphate, and guanosine 5′-monophosphate (5′-GMP) in the TAp73−/− cells (Figure 3l-3n), although only the increment in IMP reached significance at the 12 hours H_2_O_2_ time point. AMP was unchanged at 3 hours, but a non-significant increase at the 6 and 12 hours H_2_O_2_ treatment time points was observed in TAp73−/− cells. In contrast, 5′-GMP levels were increased throughout the H_2_O_2_ treatments in TAp73−/− cells, but these changes never reached significance. The changes in IMP and AMP were greatest at 12 hours H_2_O_2_, while the majority of the previously discussed purine catabolites were increased the greatest at 3 hours. Thus, the changes in IMP and AMP may represent either increased purine salvage or biosynthesis to compensate for the increased purine catabolism in the TAp73−/− cells following H_2_O_2_ treatment. The trending increases in pyrophosphate (PPi) (Figure 3o), which is formed from phosphoribosyl pyrophosphate (PRPP) during salvage and biosynthesis, and the purine biosynthesis intermediate adenylosuccinate potentially support a late increase in both purine salvage and biosynthesis in TAp73−/− cells (Figure 3p). Nonetheless, we cannot formally rule out that RNA breakdown could contribute to changes in the nucleotide pool.

In contrast to what was observed in TAp73−/− cells, IMP levels were significantly elevated only at the later H_2_O_2_ time points in WT cells, but PPi, adenylosuccinate, and AMP levels trended downwards in the WT cells. Thus, there does not appear to be increased purine salvage or biosynthesis in the WT cells, and the increase in IMP may rather represent enhanced purine catabolism at a later H_2_O_2_ time point in WT cells compared to TAp73−/− cells.

### Pyrimidine associated metabolites

**Figure 4:**
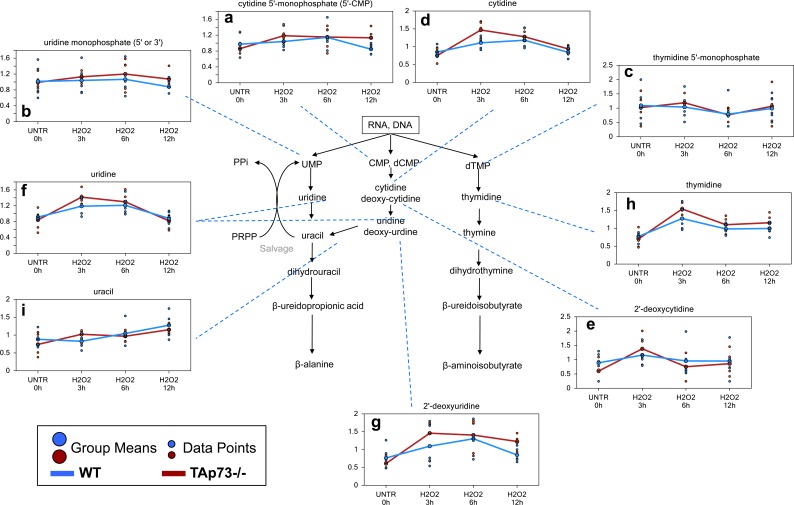
Pyrimidine associated metabolites **a.**-**i.** Levels of the indicated metabolites were evaluated as described in material and methods. Anova contrasts *t*-tests were used to identify biochemicals that differed significantly between experimental groups (*n* = 5 for each time point).

A number of pyrimidine catabolites, likely associated with increased DNA and RNA breakdown, were increased following H_2_O_2_ treatment in both WT and TAp73−/− cells and these changes were in general greatest at the 3 hour time point and in the TAp73−/− cells. These changes included early increases in the pyrimidine nucleotides (cytidine 5′-monophosphate, uridine monophosphate, and thymidine 5′monophosphate) (Figure 4a-4c), and nucleosides (cytidine, 2′-deoxycytidine, uridine, 2′-deoxyuridine, and thymidine) and the pyrimidine base (uracil) (Figure 4d-4i). Although PPi is also generated by pyrimidine salvage and/or biosynthesis, the observed changes in pyrimidine-associated metabolites do not indicate this is occurring. Rather, the observed changes suggest increased pyrimidine catabolism following H_2_O_2_ treatment, and this is more severe in the TAp73−/− cells.

### Difference in sphingosine levels may reflect decreased ceramide in TAp73−/− cells

**Figure 5:**
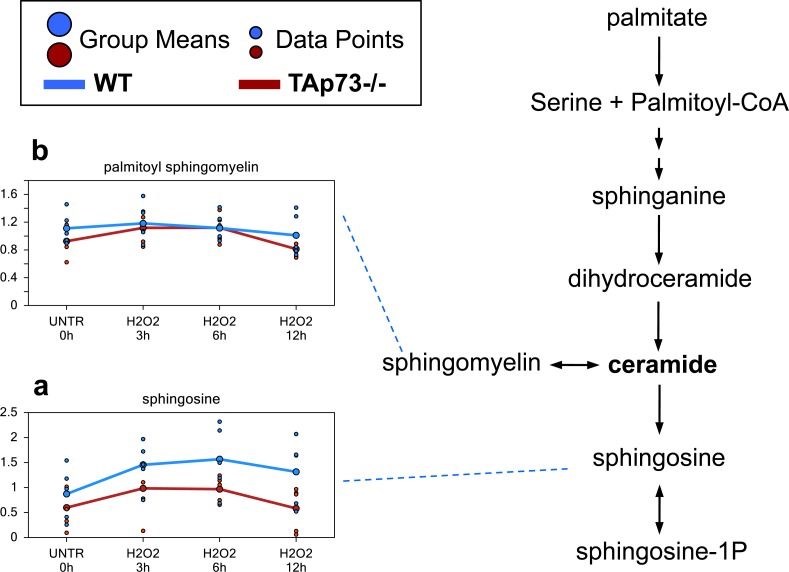
Sphingosine and ceramide metabolism Ceramide is a sphingolipid which functions as bioactive signaling molecule. Ceramide plays key roles in a variety of cellular responses, including regulation of cell growth, viability, differentiation, and senescence. **a.**-**b.** Levels of the indicated metabolites were evaluated as described in material and methods. Anova contrasts *t*-tests were used to identify biochemicals that differed significantly between experimental groups (*n* = 5 for each time point).

Increases in ceramide have been associated with growth arrest, differentiation, senescence, and H_2_O_2_-induced apoptosis [70–72]. Although we failed to detected ceramide directly, its metabolite sphingosine had a trending increase at the 3 hours H_2_O_2_ treatment time point and was significantly increased at 6 hours in both WT and TAp73−/− cells (Figure 5a). At the 12 hours, the increase in sphingosine was maintained only in the WT cells, while the levels went back to control levels in TAp73−/− cells. Furthermore, overall levels of sphingosine were consistently lower in TAp73−/− cells compared to WT cells at any time point investigated. The differences in sphingosine levels suggest increased ceramide in WT cells compared to TAp73−/− cells following H_2_O_2_ treatment. The reason for this increase in sphingosine remains unclear. Indeed, although H_2_O_2_ is known to increase sphingomyelinase activity and *de novo* ceramide synthesis, we did not observe changes in palmitoyl sphingomyelin, hence excluding increased sphingomyelinase activity. Therefore, we believe that *de novo* synthesis is the most reasonable explanation for the higher sphingosine levels (Figure 5b).

### Glycolysis is increased early only in TAp73−/− cells following H_2_O_2_ treatment

**Figure 6:**
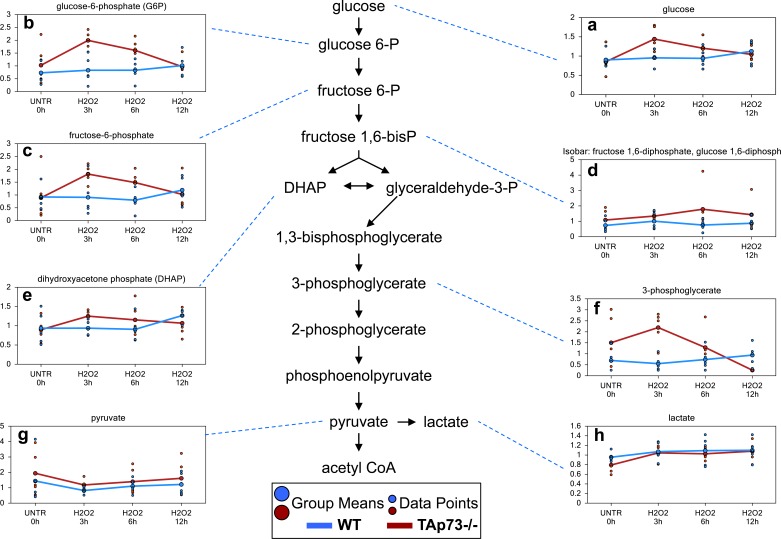
Glycolysis is increased early only in TAp73−/−following H_2_O_2_ treatment Glycolysis is the metabolic pathway that converts glucose into pyruvate. **a.**-**h.** Levels of the indicated metabolites were evaluated as described in material and methods. Anova contrasts *t*-tests were used to identify biochemicals that differed significantly between experimental groups (*n* = 5 for each time point).

One of the biggest differences between WT and TAp73−/− cells following H_2_O_2_ treatment was observed in glucose metabolism. Upon cell entry, glucose is immediately phosphorylated to glucose-6-phosphate which then can either be shunted to the PPP for NADPH production and nucleotide biosynthesis or continue through glycolysis generating pyruvate and subsequently acetyl-CoA to supply to the tricarboxylic acid (TCA) cycle for oxidative energy metabolism. We identified concerted, albeit non-significant, increases in glycolytic intermediates glucose-6-phosphate, fructose-6-phosphate, fructose 1,6-diphosphate (observed as an isobar with glucose 1,6-diphosphate), dihydroxyacetone phosphate and 3-phosphoglycerate (Figure 6a-6f) in WT H_2_O_2_-treated cells compared to WT UNTR cells at the 12 hour time point. In contrast, glucose and the glycolytic intermediates were increased earlier in the TAp73−/− cells at 3 and 6 hours, but returned to or fell below UNTR levels at 12 hour H_2_O_2_ treatment. Notwithstanding these changes, pyruvate levels in both WT and TAp73−/− (Figure 6g) cells were lower at the 3 hour treatment compared to their respective UNTR cells and subsequently began to trend upwards back to UNTR levels following 6 and 12 hour H_2_O_2_ treatments in both WT and TAp73−/−. In summary, H_2_O_2_ treatment results in an early response in TAp73−/− cells with enhanced glycolysis, while this activity is unchanged until 12 hours following H_2_O_2_ treatment in WT cells.

### Increased glycolysis induced by H_2_O_2_ in TAp73−/− cells sustains enhanced PPP

**Figure 7:**
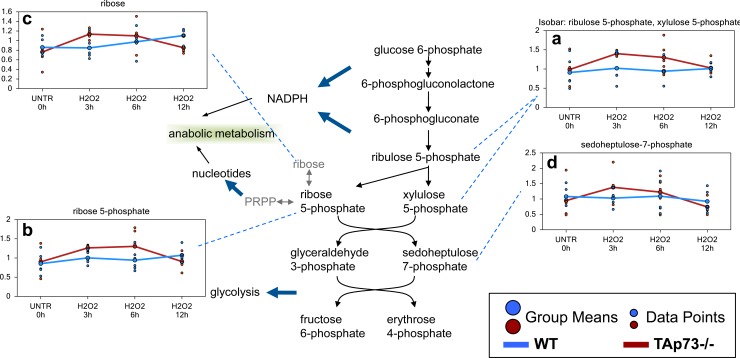
Enhanced PPP in TAp73−/− MEF after H_2_O_2_ treatment The PPP is a metabolic pathway that generates NADPH and ribose for nucleotide biosynthesis. **a.**-**d.** Levels of the indicated metabolites were evaluated as described in material and methods. Anova contrasts *t*-tests were used to identify biochemicals that differed significantly between experimental groups (*n* = 5 for each time point).

As previously discussed, one potential consequence of an early increase in glycolysis in the TAp73−/− cells following H_2_O_2_ treatment could be an increase in the PPP for NADPH production and nucleotide biosynthesis. Through sequential enzymatic steps, glucose 6-phosphate can be metabolized to ribulose 5-phosphate (observed as an isobar with xylulose 5-phosphate). Ribulose 5-phosphate is subsequently processed to ribose 5-phosphate, which can be metabolized to the end product ribose or to PRPP for nucleotide synthesis. All these metabolites were increased in TAp73−/− MEFs at 3 hour and 6 hour treatment (Figure 7a-7c). The PPP-associated biochemical sedoheptulose 7-phosphate was also increased in early H_2_O_2_-treated TAp73−/− cells, although these changes did not reach significance (Figure 7d). The changes clearly indicate that increased glycolysis in TAp73−/− cells following H_2_O_2_ treatment results in increased PPP activity that does not occur in WT cells.

### Urea cycle associated biochemicals are altered in cells treated with H_2_O_2_

**Figure 8:**
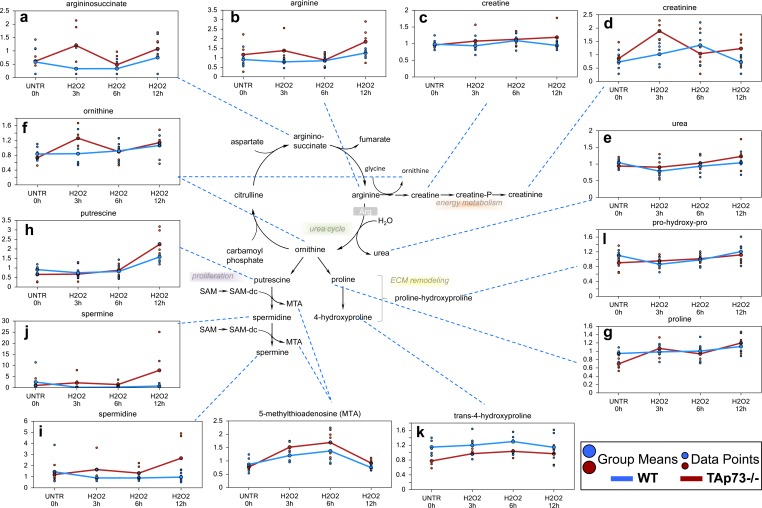
Urea cycle In the urea cycle, ornithine combines with ammonia to form citrulline. Then, a second amino group is transferred to citrulline from aspartate to form arginine the immediate precursor of urea. Arginine is hydrolyzed to urea and ornithine; thus ornithine is regenerated in each turn of the cycle. **a.**-**l.** Levels of the indicated metabolites were evaluated as described in material and methods. Anova contrasts *t*-tests were used to identify biochemicals that differed significantly between experimental groups (*n* = 5 for each time point).

We identified changes in urea cycle-associated biochemicals compatible with anaplerotic production of TCA cycle intermediates fumarate. The urea cycle functions to convert toxic ammonia to urea during amino acid catabolism [73]. The amino acid aspartate enters the urea cycle by condensing with citrulline to produce argininosuccinate. Argininosuccinate is then cleaved to form fumarate and arginine. In general, both significant and trending increases in aspartate, argininosuccinate and arginine (Figure 8a and 8b) were observed in WT and TAp73−/− cells upon H_2_O_2_ treatment, which correlated with significant increased levels of fumarate and malate (all H_2_O_2_ treatment time points in TAp73−/− cells and only 12 hour H_2_O_2_ time point in WT cells). Arginine can be further metabolized to creatine, which can be phosphorylated to creatine-phosphate, an energy storage compound. Interestingly, TAp73−/− cells showed trending increase in creatine levels over the H_2_O_2_ treatment time points that reached significance at 12 hours, an effect not observed in WT cells (Figure 8c). Changes in the levels of the spontaneous creatine-phosphate breakdown product, creatinine, also were observed during various time points of the H_2_O_2_ treatment time course (Figure 8d). The exact consequence of these changes is not clear, but an intriguing possibility is that creatine phosphate metabolism in both WT and TAp73−/− cells following H_2_O_2_ treatment acts as a potential survival mechanism.

Since ornithine is also produced during the metabolism of arginine to creatine, this may account for the increase in ornithine at the earlier time points in TAp73−/− cells, even though urea was unchanged. Ornithine can be further metabolized to proline (Figure 8g), whose metabolites contribute to synthesis of collagen or to the polyamines (putrescine, spermidine and spermine) (Figure 8h-8j). While no changes were observed in metabolites associated with extracellular matrix remodeling and collagen breakdown (proline, trans-4-hydroxyproline or pro-hydroxy-proline) in WT cells, all three of these biochemicals were significantly increased following H_2_O_2_ treatment in TAp73−/− cells depending upon the time point investigated (Figure 8k and 8l). In addition, the proliferation-associated polyamines were increased in the TAp73−/− cells over the H_2_O_2_ treatment time course and these increases reached significance at 12 hours. Overall, these changes in urea cycle metabolites in WT and TAp73−/− cells suggest that oxidative stress caused an early increase in biochemicals associated with the urea cycle in TAp73−/− cells that were not observed in WT cells. Such changes in TAp73−/− cells supports enhanced energy metabolism and anabolic activity in these cells.

## DISCUSSION

p73, together with p63 and p53, belongs to the well-established p53 gene family of transcription factors. Of these, p53 was discovered almost 40 years ago and still remains one of the most intensively studied tumor suppressor genes; as a consequence it shows very diverse, complex and articulated physiological functions, spanning from regulation of apoptosis, autophagy, mitochondria activity and oxygen radical homeostasis metabolism, DNA damage and repair pathways, maintenance of stem cell repertoire, as well as cell lineage determination [74–93]. Despite all these years of exciting investigations, many controversial issues remain to be fully clarified to elucidate the physiological and pathological roles of the p53. This wide complexity raises from different aspects and facts, including regulation by proteasomal degradation [54, 94–98] and micro-RNA [99–107] or the existence of numerous splicing variants [108–116]. Accordingly, significant efforts are under way to harness its potential practical application for human diseases, especially with regard to cancer [117–126]. On the other hand, p63 and p73 were discovered only circa 15 years ago [127–130], but already show a complexity comparable to p53, as well as a fascinating intricate interaction with p53 itself [49, 131–135]. Importantly, a certain degree of specificity characterizes p63 and p73. Indeed, p63 is pivotal for epidermal formation and homeostasis [136–144], as well as playing a role in cancer and metastasis [133, 145–156], and fertility [157–159], whereas p73 has peculiar roles in neuronal development [160–164] and fertility [165–168]. Additionally, TAp73 is a known tumor suppressor gene that regulates cell cycle progression, survival, genomic stability, hypoxia and angiogenesis [42, 45, 166, 169–178]

Numerous accruing findings indicate that TAp73 can also regulate cell metabolism [59, 61, 179–183]. Indeed, we recently showed that ectopic expression of TAp73 increases rate of glycolysis, and stimulated amino acid uptake, nucleotide biosynthesis and biosynthesis of acetyl-CoA [182, 183]. In addition, TAp73 plays an important role in the maintenance of redox homeostasis either by directly regulating the expression of the mitochondrial complex IV subunit cytochrome C oxidase subunit 4 (*COX4I1*) or by enhancing the PPP flux and hence NADPH biosynthesis [59–61]. Prompted by these findings, we sought to broaden our investigation onto whether TAp73-mediated regulation of metabolism contributes to the orchestration of cellular responses to external oxidative stress. Toward this end, we exposed TAp73 knockout and control MEFs to H_2_O_2_-mediated oxidative damage and assessed metabolic changes over a 12h time course. Overall, this study shows that a number of biochemical pathways are significantly altered following H_2_O_2_ treatment. While H_2_O_2_ induces a plausible oxidative stress response in both WT and TAp73−/− cells, the degree of response appears to be greater in TAp73−/− cells, suggesting increased susceptibility to oxidative stress in TAp73−/− cells, as previously demonstrated [59]. Notwithstanding this evidence, TAp73−/− cells probably decrease biochemicals associated with apoptosis (as demonstrated by sphingosine metabolism). Moreover, TAp73−/− cells shows changes in glucose metabolism and amino acid metabolism at earlier time points than WT cells, which may not only allow the cells to handle the oxidative stress through increased NADPH production, but may also result in pro-anabolic activity in the TAp73−/− cells. The increase in ribulose 5-phosphate/xylulose 5-phosphate in the TAp73−/− cells compare to WT cells, may suggest that an increased pool of NADPH was available to reduce glutathione. In addition, the early increases in ribose 5-phosphate and ribose in TAp73−/− following H_2_O_2_ treatment may indicate both an increase in the metabolism of ribose 5-phosphate to ribose, but also an increased capacity for nucleotide biosynthesis. This finding deserves further investigation, but, in any case, the observed changes clearly indicate that increased glycolysis in TAp73−/− cells following H_2_O_2_ treatment boosts PPP activity, an adaptation not occurring in WT cells. Intriguingly, despite the increased PPP flux should lead to enhanced NADPH synthesis and therefore higher GSH levels, we failed to detect any increase in the reduced glutathione pool in TAp73−/− MEFs. The reason for this is unclear and an accurate measurement of NADPH/NADP dynamics in these cells might help explaining this apparent conundrum. Moreover, it is also possible that the severe oxidative environment caused by exposure to H_2_O_2_ might have blunted any change in GSH or that the reduced pool of cysteine in TAp73−/− cells could have limited glutathione biosynthesis compared to their WT counterparts. Interestingly, with regard to cysteine, we observed a higher increment triggered by H_2_O_2_ treatment in TAp73−/− cells compare to WT (Figures 1 and 2). It is tempting to argue that such increase could be fueled by the glycolytic intermediate 3-phosphoglycerate (3-PG). Indeed, 3-PG is used to produce serine *via* the reaction catalyzed by 3-PG dehydrogenase, phosphoserine aminotransferase and phosphoserine phosphatase. In turn, serine can produce cysteine *via* homocysteine. Homocysteine can be the precursor of cysteine in a two-step reaction, first the condensation between homocysteine and serine catalyzed by cystathionine-β-synthase, followed by cystathionine γ-lyase-mediated production of cysteine, ammonia, and α-ketobutyrate. This attempt to compensate for the reduce cysteine levels in response to oxidative damage might contribute to the dampened glycolytic flux observed in TAp73−/− cells.

We also observed changes in nucleotide metabolism compatible with increased DNA and RNA breakdown that is potentially a consequence of oxidative damage. Once again, this increase in nucleotide breakdown appears to be more severe in the TAp73−/− cells.

In summary, our results suggest that metabolic changes in TAp73−/− cells following H_2_O_2_ treatment may result in a pro-growth metabolic profile of cells that have undergone severe oxidative damage, rather than in promotion of a cell death response under these conditions. Hence, loss of TAp73 leads, at least under oxidative stress conditions, to a rewiring of the cellular metabolism that partially resembles metabolic changes observed in cancer cells [2, 184–188], such as increase of PPP flux. The findings presented here reinforce the role of TAp73 as tumor suppressor gene and indicate that the regulation of cellular metabolism by TAp73 contributes to its tumor suppressor function. It is also fascinating to speculate that such metabolic regulations might play a role in the p53-family regulation of stem cells, as described by several research groups [47, 49, 141, 163, 164, 189–195]. Similarly, recent findings, linking epithelial-mesenchymal transition to nucleotide catabolism [196], open additional scenarios whereby regulation of nucleotide metabolism, so prominent for p73, might regulate additional cancer-related phenotypes. These and other hypotheses await investigation and could be easily tested with the use of genetically modified animals or through the flourishing CRISPR/Cas9 technology [197–199].

## MATERIALS AND METHODS

### Mice

Generation and genotype protocol of TAp73 knock-out mice were described elsewhere [166]. Mice were bred and subjected to listed procedures under the Project License released from the UK Home Office. The experimental design met the standards required by the UK Coordinating Committee on Cancer Research guidelines [200].

### Cell culture

Mouse embryonic fibroblasts (MEFs) were prepared as previously described [59]. Briefly MEFs were isolated from E13.5 littermate embryos and cultured in Dulbecco's modified Eagle's medium (DMEM) supplemented with 10% fetal calf serum, 2mM L-glutamine. Cells were treated with 0.25mM H_2_O_2_ for the indicated time.

All experiments were performed within the first 3 passages from MEFs generation to avoid ensuing senescence in primary mouse fibroblasts.

### Metabolic analysis

Sample preparation

Cells were harvested after the treatment and cell pellet stored at −80°C. Sample preparation was conducted using a proprietary series of organic and aqueous extractions to remove the protein fraction while allowing maximum recovery of small molecules. The resulting extract was divided into two fractions; one for analysis by LC and one for analysis by GC. The organic solvent was removed using a TurboVap^®^ (Zymark). Each sample was then frozen and dried under vacuum.

### Liquid chromatography/mass spectrometry (LC/MS)

Samples were then prepared for the appropriate instrument, either LC/MS or GC/MS. The sample extract was split into two aliquots, dried and then reconstituted in acidic or basic LC-compatible solvents. One aliquot was analyzed using acidic positive ion optimized conditions and the other using basic negative ion optimized conditions in two independent injections using separate dedicated columns. Extracts reconstituted in acidic conditions were gradient eluted using water and methanol both containing 0.1% Formic acid, while the basic extracts, which also used water/methanol, contained 6.5mM Ammonium Bicarbonate. The MS analysis alternated between MS and data-dependent MS^2^ scans using dynamic exclusion. The LC/MS portion of the platform was based on a Waters ACQUITY UPLC and a Thermo-Finnigan LTQ mass spectrometer, which consisted of an electrospray ionization (ESI) source and linear ion-trap mass analyzer.

Gas chromatography/mass spectrometry (GC/MS)

The samples for GC/MS analysis were re-dried under vacuum desiccation for a minimum of 24 hours prior to being derivatized under dried nitrogen using bistrimethyl-silyl-triflouroacetamide (BSTFA). The GC column was 5% phenyl and the temperature ramp is from 40° to 300°C in a 16 minute period. Samples were analyzed on a Thermo-Finnigan Trace DSQ fast-scanning single-quadrupole mass spectrometer using electron impact ionization.

### Compound identification

Compounds were identified by comparison to library entries of purified standards or recurrent unknown entities. Identification of known chemical entities was based on comparison to metabolomic library entries of purified standards. The combination of chromatographic properties and mass spectra gave an indication of a match to the specific compound or an isobaric entity.

### Statistical analysis

For these studies we perform various ANOVA procedures (e.g., repeated measures ANOVA). All results with p < 0.05 was considered significant.

## SUPPLEMENTARY MATERIALS TABLES



## Abbreviations

TAp73: Transcriptionally active p73
DMEM: Dulbecco minimal essential medium
FBS: fetal bovine serum
GC: Gas chromatography
MS: Mass spectrometry
LC: Liquid chromatography

## References

[1] Hayashi G, Cortopassi G (2015). Oxidative stress in inherited mitochondrial diseases. Free radical biology & medicine.

[2] Park MT, Kim MJ, Suh Y, Kim RK, Kim H, Lim EJ, Yoo KC, Lee GH, Kim YH, Hwang SG, Yi JM, Lee SJ (2014). Novel signaling axis for ROS generation during K-Ras-induced cellular transformation. Cell death and differentiation.

[3] Ogrunc M, Di Micco R, Liontos M, Bombardelli L, Mione M, Fumagalli M, Gorgoulis VG, d'Adda di Fagagna F (2014). Oncogene-induced reactive oxygen species fuel hyperproliferation and DNA damage response activation. Cell death and differentiation.

[4] Gorrini C, Harris IS, Mak TW (2013). Modulation of oxidative stress as an anticancer strategy. Nature reviews Drug discovery.

[5] Janssen-Heininger YM, Mossman BT, Heintz NH, Forman HJ, Kalyanaraman B, Finkel T, Stamler JS, Rhee SG, van der Vliet A (2008). Redox-based regulation of signal transduction: principles, pitfalls, and promises. Free radical biology & medicine.

[6] Agostini M, Di Marco B, Nocentini G, Delfino DV (2002). Oxidative stress and apoptosis in immune diseases. Int J Immunopathol Pharmacol.

[7] Yan F, Wang Y, Wu X, Peshavariya HM, Dusting GJ, Zhang M, Jiang F (2014). Nox4 and redox signaling mediate TGF-beta-induced endothelial cell apoptosis and phenotypic switch. Cell death & disease.

[8] Weinberg F, Hamanaka R, Wheaton WW, Weinberg S, Joseph J, Lopez M, Kalyanaraman B, Mutlu GM, Budinger GR, Chandel NS Mitochondrial metabolism and ROS generation are essential for Kras-mediated tumorigenicity.

[9] Singer E, Judkins J, Salomonis N, Matlaf L, Soteropoulos P, McAllister S, Soroceanu L (2015). Reactive oxygen species-mediated therapeutic response and resistance in glioblastoma. Cell death & disease.

[10] Harris IS, Treloar AE, Inoue S, Sasaki M, Gorrini C, Lee KC, Yung KY, Brenner D, Knobbe-Thomsen CB, Cox MA, Elia A, Berger T, Cescon DW, Adeoye A, Brustle A, Molyneux SD (2015). Glutathione and thioredoxin antioxidant pathways synergize to drive cancer initiation and progression. Cancer cell.

[11] Vallelian F, Deuel JW, Opitz L, Schaer CA, Puglia M, Lonn M, Engelsberger W, Schauer S, Karnaukhova E, Spahn DR, Stocker R, Buehler PW, Schaer DJ (2015). Proteasome inhibition and oxidative reactions disrupt cellular homeostasis during heme stress. Cell death and differentiation.

[12] Maryanovich M, Gross A (2013). A ROS rheostat for cell fate regulation. Trends in cell biology.

[13] Caputa G, Zhao S, Criado AE, Ory DS, Duncan JG, Schaffer JE (2015). RNASET2 is required for ROS propagation during oxidative stress-mediated cell death. Cell death and differentiation.

[14] Wei W, Lu Y, Hao B, Zhang K, Wang Q, Miller AL, Zhang LR, Zhang LH, Yue J (2015). CD38 Is Required for Neural Differentiation of Mouse Embryonic Stem Cells by Modulating Reactive Oxygen Species. Stem Cells.

[15] Hoarau E, Chandra V, Rustin P, Scharfmann R, Duvillie B (2014). Pro-oxidant/antioxidant balance controls pancreatic beta-cell differentiation through the ERK1/2 pathway. Cell Death Dis.

[16] Prozorovski T, Schneider R, Berndt C, Hartung HP, Aktas O (2015). Redox-regulated fate of neural stem progenitor cells. Biochim Biophys Acta.

[17] Jacquemin G, Margiotta D, Kasahara A, Bassoy EY, Walch M, Thiery J, Lieberman J, Martinvalet D (2015). Granzyme B-induced mitochondrial ROS are required for apoptosis. Cell death and differentiation.

[18] Murakami S, Motohashi H (2015). Roles of NRF2 in cell proliferation and differentiation. Free Radic Biol Med.

[19] Sela M, Tirza G, Ravid O, Volovitz I, Solodeev I, Friedman O, Zipori D, Gur E, Krelin Y, Shani N (2015). NOX1-induced accumulation of reactive oxygen species in abdominal fat-derived mesenchymal stromal cells impinges on long-term proliferation. Cell Death Dis.

[20] Okoh VO, Garba NA, Penney RB, Das J, Deoraj A, Singh KP, Sarkar S, Felty Q, Yoo C, Jackson RM, Roy D (2015). Redox signalling to nuclear regulatory proteins by reactive oxygen species contributes to oestrogen-induced growth of breast cancer cells. Br J Cancer.

[21] Hsu YC, Huang TY, Chen MJ (2014). Therapeutic ROS targeting of GADD45gamma in the induction of G2/M arrest in primary human colorectal cancer cell lines by cucurbitacin E. Cell Death Dis.

[22] Rhee SG (2006). Cell signaling. H2O2, a necessary evil for cell signaling. Science.

[23] Olsen LF, Issinger OG, Guerra B (2013). The Yin and Yang of redox regulation. Redox Rep.

[24] Pani G, Galeotti T, Chiarugi P (2010). Metastasis: cancer cell's escape from oxidative stress. Cancer metastasis reviews.

[25] Tormos KV, Anso E, Hamanaka RB, Eisenbart J, Joseph J, Kalyanaraman B, Chandel NS (2011). Mitochondrial complex III ROS regulate adipocyte differentiation. Cell metabolism.

[26] Owusu-Ansah E, Banerjee U (2009). Reactive oxygen species prime Drosophila haematopoietic progenitors for differentiation. Nature.

[27] Mikhed Y, Gorlach A, Knaus UG, Daiber A (2015). Redox regulation of genome stability by effects on gene expression, epigenetic pathways and DNA damage/repair. Redox biology.

[28] Colin DJ, Limagne E, Ragot K, Lizard G, Ghiringhelli F, Solary E, Chauffert B, Latruffe N, Delmas D (2014). The role of reactive oxygen species and subsequent DNA-damage response in the emergence of resistance towards resveratrol in colon cancer models. Cell death & disease.

[29] Liu J, Cao L, Chen J, Song S, Lee IH, Quijano C, Liu H, Keyvanfar K, Chen H, Cao LY, Ahn BH, Kumar NG, Rovira, Xu XL, van Lohuizen M, Motoyama N (2009). Bmi1 regulates mitochondrial function and the DNA damage response pathway. Nature.

[30] Neumann CA, Krause DS, Carman CV, Das S, Dubey DP, Abraham JL, Bronson RT, Fujiwara Y, Orkin SH, Van Etten RA (2003). Essential role for the peroxiredoxin Prdx1 in erythrocyte antioxidant defence and tumour suppression. Nature.

[31] Sahin E, Colla S, Liesa M, Moslehi J, Muller FL, Guo M, Cooper M, Kotton D, Fabian AJ, Walkey C, Maser RS, Tonon G, Foerster F, Xiong R, Wang YA, Shukla SA (2011). Telomere dysfunction induces metabolic and mitochondrial compromise. Nature.

[32] Schlegel CR, Georgiou ML, Misterek MB, Stocker S, Chater ER, Munro CE, Pardo OE, Seckl MJ, Costa-Pereira AP (2015). DAPK2 regulates oxidative stress in cancer cells by preserving mitochondrial function. Cell death & disease.

[33] Perales-Clemente E, Folmes CD, Terzic A (2014). Metabolic regulation of redox status in stem cells. Antioxidants & redox signaling.

[34] Itsumi M, Inoue S, Elia AJ, Murakami K, Sasaki M, Lind EF, Brenner D, Harris IS, Chio, Afzal S, Cairns RA, Cescon DW, Elford AR, Ye J, Lang PA, Li WY (2015). Idh1 protects murine hepatocytes from endotoxin-induced oxidative stress by regulating the intracellular NADP(+)/NADPH ratio. Cell death and differentiation.

[35] Ye ZW, Zhang J, Townsend DM, Tew KD (2015). Oxidative stress, redox regulation and diseases of cellular differentiation. Biochimica et biophysica acta.

[36] Srivastava S, Sinha D, Saha PP, Marthala H, D'silva P (2014). Magmas functions as a ROS regulator and provides cytoprotection against oxidative stress-mediated damages. Cell death & disease.

[37] Kalinina EV, Chernov NN, Novichkova MD (2014). Role of glutathione, glutathione transferase, and glutaredoxin in regulation of redox-dependent processes. Biochemistry Biokhimiia.

[38] Rocha CR, Garcia CC, Vieira DB, Quinet A, de Andrade-Lima LC, Munford V, Belizario JE, Menck CF (2015). Glutathione depletion sensitizes cisplatin- and temozolomide-resistant glioma cells in vitro and in vivo. Cell death & disease.

[39] Klotz LO, Sanchez-Ramos C, Prieto-Arroyo I, Urbanek P, Steinbrenner H, Monsalve M (2015). Redox regulation of FoxO transcription factors. Redox biology.

[40] Jiang L, Hickman JH, Wang SJ, Gu W (2015). Dynamic roles of p53-mediated metabolic activities in ROS-induced stress responses. Cell cycle.

[41] Gorrini C, Baniasadi PS, Harris IS, Silvester J, Inoue S, Snow B, Joshi PA, Wakeham A, Molyneux SD, Martin B, Bouwman P, Cescon DW, Elia AJ, Winterton-Perks Z, Cruickshank J, Brenner D (2013). BRCA1 interacts with Nrf2 to regulate antioxidant signaling and cell survival. The Journal of experimental medicine.

[42] Candi E, Agostini M, Melino G, Bernassola F (2014). How the TP53 Family Proteins TP63 and TP73 Contribute to Tumorigenesis: Regulators and Effectors. Human mutation.

[43] Soussi T, Wiman KG (2015). TP53: an oncogene in disguise. Cell death and differentiation.

[44] Dotsch V, Bernassola F, Coutandin D, Candi E, Melino G (2010). p63 and p73, the ancestors of p53. Cold Spring Harbor perspectives in biology.

[45] Fernandez-Alonso R, Martin-Lopez M, Gonzalez-Cano L, Garcia S, Castrillo F, Diez-Prieto I, Fernandez-Corona A, Lorenzo-Marcos ME, Li X, Claesson-Welsh L, Marques MM, Marin MC (2015). p73 is required for endothelial cell differentiation, migration and the formation of vascular networks regulating VEGF and TGFbeta signaling. Cell Death Differ.

[46] Lee HJ, Kim JM, Kim KH, Heo JI, Kwak SJ, Han JA (2015). Genotoxic stress/p53-induced DNAJB9 inhibits the pro-apoptotic function of p53. Cell death and differentiation.

[47] Memmi EM, Sanarico AG, Giacobbe A, Peschiaroli A, Frezza V, Cicalese A, Pisati F, Tosoni D, Zhou H, Tonon G, Antonov A, Melino G, Pelicci PG, Bernassola F p63 Sustains self-renewal of mammary cancer stem cells through regulation of Sonic Hedgehog signaling.

[48] Zaccara S, Tebaldi T, Pederiva C, Ciribilli Y, Bisio A, Inga A (2014). p53-directed translational control can shape and expand the universe of p53 target genes. Cell death and differentiation.

[49] Fatt MP, Cancino GI, Miller FD, Kaplan DR (2014). p63 and p73 coordinate p53 function to determine the balance between survival, cell death, and senescence in adult neural precursor cells. Cell death and differentiation.

[50] Zambetti GP (2014). Expanding the reach of the p53 tumor suppressor network. Cell death and differentiation.

[51] Shi Y, Nikulenkov F, Zawacka-Pankau J, Li H, Gabdoulline R, Xu J, Eriksson S, Hedstrom E, Issaeva N, Kel A, Arner ES, Selivanova G (2014). ROS-dependent activation of JNK converts p53 into an efficient inhibitor of oncogenes leading to robust apoptosis. Cell death and differentiation.

[52] Fitzgerald AL, Osman AA, Xie TX, Patel A, Skinner H, Sandulache V, Myers JN (2015). Reactive oxygen species and p21Waf1/Cip1 are both essential for p53-mediated senescence of head and neck cancer cells. Cell death & disease.

[53] Boudreau HE, Casterline BW, Burke DJ, Leto TL (2014). Wild-type and mutant p53 differentially regulate NADPH oxidase 4 in TGF-beta-mediated migration of human lung and breast epithelial cells. British journal of cancer.

[54] Peuget S, Bonacci T, Soubeyran P, Iovanna J, Dusetti NJ (2014). Oxidative stress-induced p53 activity is enhanced by a redox-sensitive TP53INP1 SUMOylation. Cell death and differentiation.

[55] Bensaad K, Tsuruta A, Selak MA, Vidal MN, Nakano K, Bartrons R, Gottlieb E, Vousden KH (2006). TIGAR, a p53-inducible regulator of glycolysis and apoptosis. Cell.

[56] Lee P, Hock AK, Vousden KH, Cheung EC (2015). p53- and p73-independent activation of TIGAR expression in vivo. Cell Death Dis.

[57] Sablina AA, Budanov AV, Ilyinskaya GV, Agapova LS, Kravchenko JE, Chumakov PM (2005). The antioxidant function of the p53 tumor suppressor. Nature medicine.

[58] Suzuki S, Tanaka T, Poyurovsky MV, Nagano H, Mayama T, Ohkubo S, Lokshin M, Hosokawa H, Nakayama T, Suzuki Y, Sugano S, Sato E, Nagao T, Yokote K, Tatsuno I, Prives C (2010). Phosphate-activated glutaminase (GLS2), a p53-inducible regulator of glutamine metabolism and reactive oxygen species. Proc Natl Acad Sci U S A.

[59] Rufini A, Niklison-Chirou MV, Inoue S, Tomasini R, Harris IS, Marino A, Federici M, Dinsdale D, Knight RA, Melino G, Mak TW (2012). TAp73 depletion accelerates aging through metabolic dysregulation. Genes & development.

[60] Jiang P, Du W, Yang X (2013). A critical role of glucose-6-phosphate dehydrogenase in TAp73-mediated cell proliferation. Cell cycle.

[61] Du W, Jiang P, Mancuso A, Stonestrom A, Brewer MD, Minn AJ, Mak TW, Wu M, Yang X (2013). TAp73 enhances the pentose phosphate pathway and supports cell proliferation. Nature cell biology.

[62] Giacobbe A, Bongiorno-Borbone L, Bernassola F, Terrinoni A, Markert EK, Levine AJ, Feng Z, Agostini M, Zolla L, Agro AF, Notterman DA, Melino G, Peschiaroli A (2013). p63 regulates glutaminase 2 expression. Cell cycle.

[63] Latina A, Viticchie G, Lena AM, Piro MC, Annicchiarico-Petruzzelli M, Melino G, Candi E (2016). DeltaNp63 targets cytoglobin to inhibit oxidative stress-induced apoptosis in keratinocytes and lung cancer. Oncogene.

[64] Ellisen LW, Ramsayer KD, Johannessen CM, Yang A, Beppu H, Minda K, Oliner JD, McKeon F, Haber DA (2002). REDD1, a developmentally regulated transcriptional target of p63 and p53, links p63 to regulation of reactive oxygen species. Molecular cell.

[65] Viticchie G, Agostini M, Lena AM, Mancini M, Zhou H, Zolla L, Dinsdale D, Saintigny G, Melino G, Candi E p63 supports aerobic respiration through hexokinase II.

[66] He Z, Agostini M, Liu H, Melino G, Simon HU (2015). p73 regulates basal and starvation-induced liver metabolism in vivo. Oncotarget.

[67] Dickinson DA, Forman HJ (2002). Cellular glutathione and thiols metabolism. Biochemical pharmacology.

[68] Lu SC (2013). Glutathione synthesis. Biochimica et biophysica acta.

[69] Finkelstein JD, Martin JJ (1984). Methionine metabolism in mammals. Distribution of homocysteine between competing pathways. The Journal of biological chemistry.

[70] Galadari S, Rahman A, Pallichankandy S, Thayyullathil F (2015). Tumor suppressive functions of ceramide: evidence and mechanisms. Apoptosis.

[71] Wu Y, Wang D, Wang X, Wang Y, Ren F, Chang D, Chang Z, Jia B (2011). Caspase 3 is activated through caspase 8 instead of caspase 9 during H2O2-induced apoptosis in HeLa cells. Cellular physiology and biochemistry.

[72] Yabu T, Shiba H, Shibasaki Y, Nakanishi T, Imamura S, Touhata K, Yamashita M (2015). Stress-induced ceramide generation and apoptosis via the phosphorylation and activation of nSMase1 by JNK signaling. Cell death and differentiation.

[73] Dimski DS (1994). Ammonia metabolism and the urea cycle: function and clinical implications. Journal of veterinary internal medicine.

[74] Ci Y, Shi K, An J, Yang Y, Hui K, Wu P, Shi L, Xu C (2014). ROS inhibit autophagy by downregulating ULK1 mediated by the phosphorylation of p53 in selenite-treated NB4 cells. Cell death & disease.

[75] Evstafieva AG, Garaeva AA, Khutornenko AA, Klepikova AV, Logacheva MD, Penin AA, Novakovsky GE, Kovaleva IE, Chumakov PM (2014). A sustained deficiency of mitochondrial respiratory complex III induces an apoptotic cell death through the p53-mediated inhibition of pro-survival activities of the activating transcription factor 4. Cell death & disease.

[76] Garufi A, Pucci D, D'Orazi V, Cirone M, Bossi G, Avantaggiati ML, D'Orazi G (2014). Degradation of mutant p53H175 protein by Zn(II) through autophagy. Cell death & disease.

[77] Rufini A, Tucci P, Celardo I, Melino G (2013). Senescence and aging: the critical roles of p53. Oncogene.

[78] Simon HU, Yousefi S, Schmid I, Friis R (2014). ATG5 can regulate p53 expression and activation. Cell Death Dis.

[79] Avkin S, Sevilya Z, Toube L, Geacintov N, Chaney SG, Oren M, Livneh Z (2006). p53 and p21 regulate error-prone DNA repair to yield a lower mutation load. Molecular cell.

[80] Dashzeveg N, Taira N, Lu ZG, Kimura J, Yoshida K (2014). Palmdelphin, a novel target of p53 with Ser46 phosphorylation, controls cell death in response to DNA damage. Cell death & disease.

[81] Gao Y, Ferguson DO, Xie W, Manis JP, Sekiguchi J, Frank KM, Chaudhuri J, Horner J, DePinho RA, Alt FW (2000). Interplay of p53 and DNA-repair protein XRCC4 in tumorigenesis, genomic stability and development. Nature.

[82] Li L, Ng DS, Mah WC, Almeida FF, Rahmat SA, Rao VK, Leow SC, Laudisi F, Peh MT, Goh AM, Lim JS, Wright GD, Mortellaro A, Taneja R, Ginhoux F, Lee CG (2015). A unique role for p53 in the regulation of M2 macrophage polarization. Cell death and differentiation.

[83] Seo YR, Fishel ML, Amundson S, Kelley MR, Smith ML (2002). Implication of p53 in base excision DNA repair: in vivo evidence. Oncogene.

[84] Nair BC, Krishnan SR, Sareddy GR, Mann M, Xu B, Natarajan M, Hasty P, Brann D, Tekmal RR, Vadlamudi RK (2014). Proline, glutamic acid and leucine-rich protein-1 is essential for optimal p53-mediated DNA damage response. Cell death and differentiation.

[85] Nicolai S, Rossi A, Di Daniele N, Melino G, Annicchiarico-Petruzzelli M, Raschella G (2015). DNA repair and aging: the impact of the p53 family. Aging (Albany NY).

[86] Phesse TJ, Myant KB, Cole AM, Ridgway RA, Pearson H, Muncan V, van den Brink GR, Vousden KH, Sears R, Vassilev LT, Clarke AR, Sansom OJ (2014). Endogenous c-Myc is essential for p53-induced apoptosis in response to DNA damage in vivo. Cell death and differentiation.

[87] Saifudeen Z, Dipp S, El-Dahr SS (2002). A role for p53 in terminal epithelial cell differentiation. The Journal of clinical investigation.

[88] Serrano MA, Li Z, Dangeti M, Musich PR, Patrick S, Roginskaya M, Cartwright B, Zou Y (2013). DNA-PK, ATM and ATR collaboratively regulate p53-RPA interaction to facilitate homologous recombination DNA repair. Oncogene.

[89] Smith ML, Seo YR (2002). p53 regulation of DNA excision repair pathways. Mutagenesis.

[90] Xu J, Wang J, Hu Y, Qian J, Xu B, Chen H, Zou W, Fang JY (2014). Unequal prognostic potentials of p53 gain-of-function mutations in human cancers associate with drug-metabolizing activity. Cell death & disease.

[91] Eby KG, Rosenbluth JM, Mays DJ, Marshall CB, Barton CE, Sinha S, Johnson KN, Tang L, Pietenpol JA (2010). ISG20L1 is a p53 family target gene that modulates genotoxic stress-induced autophagy. Molecular cancer.

[92] Tasdemir E, Maiuri MC, Galluzzi L, Vitale I, Djavaheri-Mergny M, D'Amelio M, Criollo A, Morselli E, Zhu C, Harper F, Nannmark U, Samara C, Pinton P, Vicencio JM, Carnuccio R, Moll UM (2008). Regulation of autophagy by cytoplasmic p53. Nature cell biology.

[93] Sui X, Jin L, Huang X, Geng S, He C, Hu X (2011). p53 signaling and autophagy in cancer: a revolutionary strategy could be developed for cancer treatment. Autophagy.

[94] Liu J, Zhang C, Wang XL, Ly P, Belyi V, Xu-Monette ZY, Young KH, Hu W, Feng Z (2014). E3 ubiquitin ligase TRIM32 negatively regulates tumor suppressor p53 to promote tumorigenesis. Cell death and differentiation.

[95] Zhang HH, Li SZ, Zhang ZY, Hu XM, Hou PN, Gao L, Du RL, Zhang XD (2014). Nemo-like kinase is critical for p53 stabilization and function in response to DNA damage. Cell death and differentiation.

[96] Sane S, Abdullah A, Boudreau DA, Autenried RK, Gupta BK, Wang X, Wang H, Schlenker EH, Zhang D, Telleria C, Huang L, Chauhan SC, Rezvani K (2014). Ubiquitin-like (UBX)-domain-containing protein, UBXN2A, promotes cell death by interfering with the p53-Mortalin interactions in colon cancer cells. Cell death & disease.

[97] Xu C, Fan CD, Wang X (2015). Regulation of Mdm2 protein stability and the p53 response by NEDD4-1 E3 ligase. Oncogene.

[98] Fu X, Yucer N, Liu S, Li M, Yi P, Mu JJ, Yang T, Chu J, Jung SY, O'Malley BW, Gu W, Qin J, Wang Y RFWD3-Mdm2 ubiquitin ligase complex positively regulates p53 stability in response to DNA damage.

[99] Mu W, Hu C, Zhang H, Qu Z, Cen J, Qiu Z, Li C, Ren H, Li Y, He X, Shi X, Hui L (2015). miR-27b synergizes with anticancer drugs via p53 activation and CYP1B1 suppression. Cell research.

[100] Leotta M, Biamonte L, Raimondi L, Ronchetti D, Di Martino MT, Botta C, Leone E, Pitari MR, Neri A, Giordano A, Tagliaferri P, Tassone P, Amodio N (2014). A p53-dependent tumor suppressor network is induced by selective miR-125a-5p inhibition in multiple myeloma cells. Journal of cellular physiology.

[101] Wang F, Lv P, Liu X, Zhu M, Qiu X (2015). microRNA-214 enhances the invasion ability of breast cancer cells by targeting p53. International journal of molecular medicine.

[102] Fortunato O, Boeri M, Moro M, Verri C, Mensah M, Conte D, Caleca L, Roz L, Pastorino U, Sozzi G (2014). Mir-660 is downregulated in lung cancer patients and its replacement inhibits lung tumorigenesis by targeting MDM2-p53 interaction. Cell death & disease.

[103] Fiori ME, Barbini C, Haas TL, Marroncelli N, Patrizii M, Biffoni M, De Maria R (2014). Antitumor effect of miR-197 targeting in p53 wild-type lung cancer. Cell death and differentiation.

[104] Hoffman Y, Bublik DR, Pilpel Y, Oren M (2014). miR-661 downregulates both Mdm2 and Mdm4 to activate p53. Cell death and differentiation.

[105] Shin S, Lee EM, Cha HJ, Bae S, Jung JH, Lee SM, Yoon Y, Lee H, Kim S, Kim H, Lee SJ, Park IC, Jin YW, An S (2009). MicroRNAs that respond to histone deacetylase inhibitor SAHA and p53 in HCT116 human colon carcinoma cells. International journal of oncology.

[106] Zhang C, Liu J, Wang X, Wu R, Lin M, Laddha SV, Yang Q, Chan CS, Feng Z (2014). MicroRNA-339-5p inhibits colorectal tumorigenesis through regulation of the MDM2/p53 signaling. Oncotarget.

[107] Ren ZJ, Nong XY, Lv YR, Sun HH, An PP, Wang F, Li X, Liu M, Tang H (2014). Mir-509-5p joins the Mdm2/p53 feedback loop and regulates cancer cell growth. Cell death & disease.

[108] Silden E, Hjelle SM, Wergeland L, Sulen A, Andresen V, Bourdon JC, Micklem DR, McCormack E, Gjertsen BT (2013). Expression of TP53 isoforms p53beta or p53gamma enhances chemosensitivity in TP53(null) cell lines. PloS one.

[109] Bernard H, Garmy-Susini B, Ainaoui N, Van Den Berghe L, Peurichard A, Javerzat S, Bikfalvi A, Lane DP, Bourdon JC, Prats AC (2013). The p53 isoform, Delta133p53alpha, stimulates angiogenesis and tumour progression. Oncogene.

[110] Bourdon JC, Fernandes K, Murray-Zmijewski F, Liu G, Diot A, Xirodimas DP, Saville MK, Lane DP (2005). p53 isoforms can regulate p53 transcriptional activity. Genes & development.

[111] Fujita K, Mondal AM, Horikawa I, Nguyen GH, Kumamoto K, Sohn JJ, Bowman ED, Mathe EA, Schetter AJ, Pine SR, Ji H, Vojtesek B, Bourdon JC, Lane DP, Harris CC (2009). p53 isoforms Delta133p53 and p53beta are endogenous regulators of replicative cellular senescence. Nature cell biology.

[112] Marcel V, Fernandes K, Terrier O, Lane DP, Bourdon JC (2014). Modulation of p53beta and p53gamma expression by regulating the alternative splicing of TP53 gene modifies cellular response. Cell death and differentiation.

[113] Marcel V, Perrier S, Aoubala M, Ageorges S, Groves MJ, Diot A, Fernandes K, Tauro S, Bourdon JC (2010). Delta160p53 is a novel N-terminal p53 isoform encoded by Delta133p53 transcript. FEBS letters.

[114] Joruiz SM, Bourdon JC (2016). p53 Isoforms: Key Regulators of the Cell Fate Decision. Cold Spring Harbor perspectives in medicine.

[115] Slatter TL, Hung N, Bowie S, Campbell H, Rubio C, Speidel D, Wilson M, Baird M, Royds JA, Braithwaite AW (2015). Delta122p53, a mouse model of Delta133p53alpha, enhances the tumor-suppressor activities of an attenuated p53 mutant. Cell death & disease.

[116] Solomon H, Sharon M, Rotter V (2014). Modulation of alternative splicing contributes to cancer development: focusing on p53 isoforms, p53beta and p53gamma. Cell Death Differ.

[117] Amelio I, Landre V, Knight RA, Lisitsa A, Melino G, Antonov AV (2015). Polypharmacology of small molecules targeting the ubiquitin-proteasome and ubiquitin-like systems. Oncotarget.

[118] Grigoreva TA, Tribulovich VG, Garabadzhiu AV, Melino G, Barlev NA (2015). The 26S proteasome is a multifaceted target for anti-cancer therapies. Oncotarget.

[119] Bykov VJ, Wiman KG (2014). Mutant p53 reactivation by small molecules makes its way to the clinic. FEBS letters.

[120] Pellegrino M, Mancini F, Luca R, Coletti A, Giacche N, Manni I, Arisi I, Florenzano F, Teveroni E, Buttarelli M, Fici L, Brandi R, Bruno T, Fanciulli M, D'Onofrio M, Piaggio G (2015). Targeting the MDM2/MDM4 Interaction Interface as a Promising Approach for p53 Reactivation Therapy. Cancer research.

[121] Soares J, Raimundo L, Pereira NA, Monteiro A, Gomes S, Bessa C, Pereira C, Queiroz G, Bisio A, Fernandes J, Gomes C, Reis F, Goncalves J, Inga A, Santos MM, Saraiva L (2016). Reactivation of wild-type and mutant p53 by tryptophanolderived oxazoloisoindolinone SLMP53-1, a novel anticancer small-molecule. Oncotarget.

[122] Hiraki M, Hwang SY, Cao S, Ramadhar TR, Byun S, Yoon KW, Lee JH, Chu K, Gurkar AU, Kolev V, Zhang J, Namba T, Murphy ME, Newman DJ, Mandinova A, Clardy J (2015). Small-Molecule Reactivation of Mutant p53 to Wild-Type-like p53 through the p53-Hsp40 Regulatory Axis. Chemistry & biology.

[123] Weilbacher A, Gutekunst M, Oren M, Aulitzky WE, van der Kuip H (2014). RITA can induce cell death in p53-defective cells independently of p53 function via activation of JNK/SAPK and p38. Cell death & disease.

[124] Cheng J, Fan YH, Xu X, Zhang H, Dou J, Tang Y, Zhong X, Rojas Y, Yu Y, Zhao Y, Vasudevan SA, Zhang H, Nuchtern JG, Kim ES, Chen X, Lu F (2014). A small-molecule inhibitor of UBE2N induces neuroblastoma cell death via activation of p53 and JNK pathways. Cell death & disease.

[125] Becker MS, Schmezer P, Breuer R, Haas SF, Essers MA, Krammer PH, Li-Weber M (2014). The traditional Chinese medical compound Rocaglamide protects nonmalignant primary cells from DNA damage-induced toxicity by inhibition of p53 expression. Cell death & disease.

[126] Rossi M, Rotblat B, Ansell K, Amelio I, Caraglia M, Misso G, Bernassola F, Cavasotto CN, Knight RA, Ciechanover A, Melino G (2014). High throughput screening for inhibitors of the HECT ubiquitin E3 ligase ITCH identifies antidepressant drugs as regulators of autophagy. Cell death & disease.

[127] Yang A, Kaghad M, Wang Y, Gillett E, Fleming MD, Dotsch V, Andrews NC, Caput D, McKeon F (1998). p63, a p53 homolog at 3q27-29, encodes multiple products with transactivating, death-inducing, and dominant-negative activities. Molecular cell.

[128] Kaghad M, Bonnet H, Yang A, Creancier L, Biscan JC, Valent A, Minty A, Chalon P, Lelias JM, Dumont X, Ferrara P, McKeon F, Caput D (1997). Monoallelically expressed gene related to p53 at 1p36, a region frequently deleted in neuroblastoma and other human cancers. Cell.

[129] Trink B, Okami K, Wu L, Sriuranpong V, Jen J, Sidransky D (1998). A new human p53 homologue. Nature medicine.

[130] Schmale H, Bamberger C (1997). A novel protein with strong homology to the tumor suppressor p53. Oncogene.

[131] Adamovich Y, Adler J, Meltser V, Reuven N, Shaul Y (2014). AMPK couples p73 with p53 in cell fate decision. Cell death and differentiation.

[132] Bunjobpol W, Dulloo I, Igarashi K, Concin N, Matsuo K, Sabapathy K (2014). Suppression of acetylpolyamine oxidase by selected AP-1 members regulates DNp73 abundance: mechanistic insights for overcoming DNp73-mediated resistance to chemotherapeutic drugs. Cell death and differentiation.

[133] Adorno M, Cordenonsi M, Montagner M, Dupont S, Wong C, Hann B, Solari A, Bobisse S, Rondina MB, Guzzardo V, Parenti AR, Rosato A, Bicciato S, Balmain A, Piccolo S (2009). A Mutant-p53/Smad complex opposes p63 to empower TGFbeta-induced metastasis. Cell.

[134] Flores ER, Tsai KY, Crowley D, Sengupta S, Yang A, McKeon F, Jacks T (2002). p63 and p73 are required for p53-dependent apoptosis in response to DNA damage. Nature.

[135] Xu J, Reumers J, Couceiro JR, De Smet F, Gallardo R, Rudyak S, Cornelis A, Rozenski J, Zwolinska A, Marine JC, Lambrechts D, Suh YA, Rousseau F, Schymkowitz J (2011). Gain of function of mutant p53 by coaggregation with multiple tumor suppressors. Nature chemical biology.

[136] Candi E, Rufini A, Terrinoni A, Dinsdale D, Ranalli M, Paradisi A, De Laurenzi V, Spagnoli LG, Catani MV, Ramadan S, Knight RA, Melino G (2006). Differential roles of p63 isoforms in epidermal development: selective genetic complementation in p63 null mice. Cell death and differentiation.

[137] Mills AA, Zheng B, Wang XJ, Vogel H, Roop DR, Bradley A (1999). p63 is a p53 homologue required for limb and epidermal morphogenesis. Nature.

[138] Botchkarev VA, Flores ER (2014). p53/p63/p73 in the epidermis in health and disease. Cold Spring Harbor perspectives in medicine.

[139] Burnley P, Rahman M, Wang H, Zhang Z, Sun X, Zhuge Q, Su DM (2013). Role of the p63-FoxN1 regulatory axis in thymic epithelial cell homeostasis during aging. Cell death & disease.

[140] Candi E, Terrinoni A, Rufini A, Chikh A, Lena AM, Suzuki Y, Sayan BS, Knight RA, Melino G (2006). p63 is upstream of IKK alpha in epidermal development. Journal of cell science.

[141] Crum CP, McKeon FD (2010). p63 in epithelial survival, germ cell surveillance, and neoplasia. Annual review of pathology.

[142] Rufini A, Weil M, McKeon F, Barlattani A, Melino G, Candi E (2006). p63 protein is essential for the embryonic development of vibrissae and teeth. Biochemical and biophysical research communications.

[143] Senoo M, Pinto F, Crum CP, McKeon F (2007). p63 Is essential for the proliferative potential of stem cells in stratified epithelia. Cell.

[144] Yang A, Schweitzer R, Sun D, Kaghad M, Walker N, Bronson RT, Tabin C, Sharpe A, Caput D, Crum C, McKeon F (1999). p63 is essential for regenerative proliferation in limb, craniofacial and epithelial development. Nature.

[145] Yallowitz AR, Alexandrova EM, Talos F, Xu S, Marchenko ND, Moll UM (2014). p63 is a prosurvival factor in the adult mammary gland during post-lactational involution, affecting PI-MECs and ErbB2 tumorigenesis. Cell death and differentiation.

[146] Salah Z, Bar-mag T, Kohn Y, Pichiorri F, Palumbo T, Melino G, Aqeilan RI (2013). Tumor suppressor WWOX binds to DeltaNp63alpha and sensitizes cancer cells to chemotherapy. Cell death & disease.

[147] Su X, Chakravarti D, Cho MS, Liu L, Gi YJ, Lin YL, Leung ML, El-Naggar A, Creighton CJ, Suraokar MB, Wistuba I, Flores ER (2010). TAp63 suppresses metastasis through coordinate regulation of Dicer and miRNAs. Nature.

[148] Wu J, Liang S, Bergholz J, He H, Walsh EM, Zhang Y, Xiao ZX (2014). DeltaNp63alpha activates CD82 metastasis suppressor to inhibit cancer cell invasion. Cell death & disease.

[149] Giacobbe A, Compagnone M, Bongiorno-Borbone L, Antonov A, Markert EK, Zhou JH, Annicchiarico-Petruzzelli M, Melino G, Peschiaroli A (2016). p63 controls cell migration and invasion by transcriptional regulation of MTSS1. Oncogene.

[150] Lee KB, Ye S, Park MH, Park BH, Lee JS, Kim SM (2014). p63-Mediated activation of the beta-catenin/c-Myc signaling pathway stimulates esophageal squamous carcinoma cell invasion and metastasis. Cancer letters.

[151] Tucci P, Agostini M, Grespi F, Markert EK, Terrinoni A, Vousden KH, Muller PA, Dotsch V, Kehrloesser S, Sayan BS, Giaccone G, Lowe SW, Takahashi N, Vandenabeele P, Knight RA, Levine AJ Loss of p63 and its microRNA-205 target results in enhanced cell migration and metastasis in prostate cancer.

[152] Cho MS, Chan IL, Flores ER (2010). DeltaNp63 transcriptionally regulates brachyury, a gene with diverse roles in limb development, tumorigenesis and metastasis. Cell cycle.

[153] Bornachea O, Lopez-Calderon FF, Duenas M, Segrelles C, Lorz C, Suarez-Cabrera C, Maranon M, Paradela-Dobarro B, Santos M, Paramio JM (2015). The downregulation of DeltaNp63 in p53-deficient mouse epidermal tumors favors metastatic behavior. Oncotarget.

[154] Srivastava K, Pickard A, McDade S, McCance DJ (2015). p63 drives invasion in keratinocytes expressing HPV16 E6/E7 genes through regulation of Src-FAK signalling. Oncotarget.

[155] Guo X, Keyes WM, Papazoglu C, Zuber J, Li W, Lowe SW, Vogel H, Mills AA (2009). TAp63 induces senescence and suppresses tumorigenesis in vivo. Nature cell biology.

[156] Keyes WM, Pecoraro M, Aranda V, Vernersson-Lindahl E, Li W, Vogel H, Guo X, Garcia EL, Michurina TV, Enikolopov G, Muthuswamy SK, Mills AA (2011). DeltaNp63alpha is an oncogene that targets chromatin remodeler Lsh to drive skin stem cell proliferation and tumorigenesis. Cell stem cell.

[157] Amelio I, Grespi F, Annicchiarico-Petruzzelli M, Melino G (2012). p63 the guardian of human reproduction. Cell cycle.

[158] Suh EK, Yang A, Kettenbach A, Bamberger C, Michaelis AH, Zhu Z, Elvin JA, Bronson RT, Crum CP, McKeon F (2006). p63 protects the female germ line during meiotic arrest. Nature.

[159] Kerr JB, Hutt KJ, Michalak EM, Cook M, Vandenberg CJ, Liew SH, Bouillet P, Mills A, Scott CL, Findlay JK, Strasser A (2012). DNA damage-induced primordial follicle oocyte apoptosis and loss of fertility require TAp63-mediated induction of Puma and Noxa. Molecular cell.

[160] Yang A, Walker N, Bronson R, Kaghad M, Oosterwegel M, Bonnin J, Vagner C, Bonnet H, Dikkes P, Sharpe A, McKeon F, Caput D (2000). p73-deficient mice have neurological, pheromonal and inflammatory defects but lack spontaneous tumours. Nature.

[161] Killick R, Niklison-Chirou M, Tomasini R, Bano D, Rufini A, Grespi F, Velletri T, Tucci P, Sayan BS, Conforti F, Gallagher E, Nicotera P, Mak TW, Melino G, Knight RA, Agostini M (2011). p73: a multifunctional protein in neurobiology. Molecular neurobiology.

[162] Agostini M, Tucci P, Killick R, Candi E, Sayan BS, Rivetti di Val Cervo P, Nicotera P, McKeon F, Knight RA, Mak TW, Melino G Neuronal differentiation by TAp73 is mediated by microRNA-34a regulation of synaptic protein targets.

[163] Niklison-Chirou MV, Killick R, Knight RA, Nicotera P, Melino G, Agostini M (2015). How Does p73 Cause Neuronal Defects?. Molecular neurobiology.

[164] Niklison-Chirou MV, Steinert JR, Agostini M, Knight RA, Dinsdale D, Cattaneo A, Mak TW, Melino G TAp73 knockout mice show morphological and functional nervous system defects associated with loss of p75 neurotrophin receptor.

[165] Inoue S, Tomasini R, Rufini A, Elia AJ, Agostini M, Amelio I, Cescon D, Dinsdale D, Zhou L, Harris IS, Lac S, Silvester J, Li WY, Sasaki M, Haight J, Brustle A TAp73 is required for spermatogenesis and the maintenance of male fertility.

[166] Tomasini R, Tsuchihara K, Wilhelm M, Fujitani M, Rufini A, Cheung CC, Khan F, Itie-Youten A, Wakeham A, Tsao MS, Iovanna JL, Squire J, Jurisica I, Kaplan D, Melino G, Jurisicova A (2008). TAp73 knockout shows genomic instability with infertility and tumor suppressor functions. Genes & development.

[167] Holembowski L, Kramer D, Riedel D, Sordella R, Nemajerova A, Dobbelstein M, Moll UM (2014). TAp73 is essential for germ cell adhesion and maturation in testis. The Journal of cell biology.

[168] Guglielmino MR, Santonocito M, Vento M, Ragusa M, Barbagallo D, Borzi P, Casciano I, Banelli B, Barbieri O, Astigiano S, Scollo P, Romani M, Purrello M, Di Pietro C (2011). TAp73 is downregulated in oocytes from women of advanced reproductive age. Cell cycle.

[169] Rosenbluth JM, Pietenpol JA (2008). The jury is in: p73 is a tumor suppressor after all. Genes & development.

[170] Wilhelm MT, Rufini A, Wetzel MK, Tsuchihara K, Inoue S, Tomasini R, Itie-Youten A, Wakeham A, Arsenian-Henriksson M, Melino G, Kaplan DR, Miller FD, Mak TW (2010). Isoform-specific p73 knockout mice reveal a novel role for delta Np73 in the DNA damage response pathway. Genes & development.

[171] Amelio I, Inoue S, Markert EK, Levine AJ, Knight RA, Mak TW, Melino G TAp73 opposes tumor angiogenesis by promoting hypoxia-inducible factor 1alpha degradation.

[172] Amelio I, Melino G (2015). The p53 family and the hypoxia-inducible factors (HIFs): determinants of cancer progression. Trends in biochemical sciences.

[173] Petrova V, Mancini M, Agostini M, Knight RA, Annicchiarico-Petruzzelli M, Barlev NA, Melino G, Amelio I (2015). TAp73 transcriptionally represses BNIP3 expression. Cell cycle.

[174] Stantic M, Sakil HA, Zirath H, Fang T, Sanz G, Fernandez-Woodbridge A, Marin A, Susanto E, Mak TW, Arsenian Henriksson M, Wilhelm MT TAp73 suppresses tumor angiogenesis through repression of proangiogenic cytokines and HIF-1alpha activity.

[175] Dulloo I, Hooi PB, Sabapathy K (2015). Hypoxia-induced DNp73 stabilization regulates Vegf-A expression and tumor angiogenesis similar to TAp73. Cell cycle.

[176] Tomasini R, Tsuchihara K, Tsuda C, Lau SK, Wilhelm M, Ruffini A, Tsao MS, Iovanna JL, Jurisicova A, Melino G, Mak TW TAp73 regulates the spindle assembly checkpoint by modulating BubR1 activity.

[177] Riley MF, You MJ, Multani AS, Lozano G (2016). Mdm2 overexpression and p73 loss exacerbate genomic instability and dampen apoptosis, resulting in B-cell lymphoma. Oncogene.

[178] Beitzinger M, Hofmann L, Oswald C, Beinoraviciute-Kellner R, Sauer M, Griesmann H, Bretz AC, Burek C, Rosenwald A, Stiewe T (2008). p73 poses a barrier to malignant transformation by limiting anchorage-independent growth. The EMBO journal.

[179] Amelio I, Markert EK, Rufini A, Antonov AV, Sayan BS, Tucci P, Agostini M, Mineo TC, Levine AJ, Melino G (2014). p73 regulates serine biosynthesis in cancer. Oncogene.

[180] Velletri T, Romeo F, Tucci P, Peschiaroli A, Annicchiarico-Petruzzelli M, Niklison-Chirou MV, Amelio I, Knight RA, Mak TW, Melino G, Agostini M (2013). GLS2 is transcriptionally regulated by p73 and contributes to neuronal differentiation. Cell cycle.

[181] Cutruzzola F, Avigliano L, Candi E (2014). p73 keeps metabolic control in balance. Cell cycle.

[182] Agostini M, Niklison-Chirou MV, Catani MV, Knight RA, Melino G, Rufini A (2014). TAp73 promotes anti-senescence-anabolism not proliferation. Aging (Albany NY).

[183] Amelio I, Antonov AA, Catani MV, Massoud R, Bernassola F, Knight RA, Melino G, Rufini A (2014). TAp73 promotes anabolism. Oncotarget.

[184] Cairns RA, Harris IS, Mak TW (2011). Regulation of cancer cell metabolism. Nature reviews Cancer.

[185] Amelio I, Cutruzzola F, Antonov A, Agostini M, Melino G (2014). Serine and glycine metabolism in cancer. Trends in biochemical sciences.

[186] Antonov A, Agostini M, Morello M, Minieri M, Melino G, Amelio I (2014). Bioinformatics analysis of the serine and glycine pathway in cancer cells. Oncotarget.

[187] Alberghina L, Gaglio D (2014). Redox control of glutamine utilization in cancer. Cell death & disease.

[188] Tian Y, Kuo CF, Sir D, Wang L, Govindarajan S, Petrovic LM, Ou JH (2015). Autophagy inhibits oxidative stress and tumor suppressors to exert its dual effect on hepatocarcinogenesis. Cell death and differentiation.

[189] Velletri T, Romeo F, Tucci P, Peschiaroli A, Annicchiarico-Petruzzelli M, Niklison-Chirou M, Amelio I, Knight R, Mak T, Melino G, Agostini M (2015). GLS2 is transcriptionally regulated by p73 and contributes to neuronal differentiation. Cell cycle.

[190] Melino G, Memmi EM, Pelicci PG, Bernassola F (2015). Maintaining epithelial stemness with p63. Science signaling.

[191] Candi E, Amelio I, Agostini M, Melino G (2015). MicroRNAs and p63 in epithelial stemness. Cell death and differentiation.

[192] Agostini M, Tucci P, Chen H, Knight RA, Bano D, Nicotera P, McKeon F, Melino G (2010). p73 regulates maintenance of neural stem cell. Biochemical and biophysical research communications.

[193] Dugani CB, Paquin A, Fujitani M, Kaplan DR, Miller FD (2009). p63 antagonizes p53 to promote the survival of embryonic neural precursor cells. The Journal of neuroscience.

[194] Fujitani M, Cancino GI, Dugani CB, Weaver IC, Gauthier-Fisher A, Paquin A, Mak TW, Wojtowicz MJ, Miller FD, Kaplan DR (2010). TAp73 acts via the bHLH Hey2 to promote long-term maintenance of neural precursors. Current biology.

[195] Talos F, Abraham A, Vaseva AV, Holembowski L, Tsirka SE, Scheel A, Bode D, Dobbelstein M, Bruck W, Moll UM (2010). p73 is an essential regulator of neural stem cell maintenance in embryonal and adult CNS neurogenesis. Cell death and differentiation.

[196] Shaul YD, Freinkman E, Comb WC, Cantor JR, Tam WL, Thiru P, Kim D, Kanarek N, Pacold ME, Chen WW, Bierie B, Possemato R, Reinhardt F, Weinberg RA, Yaffe MB, Sabatini DM (2014). Dihydropyrimidine accumulation is required for the epithelial-mesenchymal transition. Cell.

[197] Vasileva EA, Shuvalov OU, Garabadgiu AV, Melino G, Barlev NA (2015). Genome-editing tools for stem cell biology. Cell death & disease.

[198] Amelio I, Melino G (2015). CRISPR: a new method for genetic engineering - a prokaryotic immune component may potentially open a new era of gene silencing. Cell death and differentiation.

[199] Doudna JA, Charpentier E (2014). Genome editing. The new frontier of genome engineering with CRISPR-Cas9. Science.

[200] Workman P, Aboagye EO, Balkwill F, Balmain A, Bruder G, Chaplin DJ, Double JA, Everitt J, Farningham DA, Glennie MJ, Kelland LR, Robinson V, Stratford IJ, Tozer GM, Watson S, Wedge SR (2010). Guidelines for the welfare and use of animals in cancer research. British journal of cancer.

