# Supplementary material for: Metabolic pathways regulated by TAp73 in response to oxidative stress
Source: Oncotarget. 2016 Apr 22;7(21):29881–900. doi: 10.18632/oncotarget.8935 (PMC5058650; doi:10.18632/oncotarget.8935)
Supplement: Supplementary file 1 [file oncotarget-07-29881-s001.pdf]

Metabolic pathways regulated by TAp73 in response to oxidative stress

Supplementary Material

Table S1. Metabolite summary and significantly altered biochemicals.

Statistical Comparisons				
**ANOVA Contrasts**	Total number of biochemicals with *p*≤0.05	Biochemicals (↑↓) p≤0.05	Total number of biochemicals with 0.05<*p*<0.10	Biochemicals (↑↓) 0.05<p<0.10
**WT 3H / WT UNTR**	33	11|22	16	5|11
**WT 6H / WT UNTR**	32	29|3	17	15|2
**WT 12H / WT UNTR**	36	17|19	20	10|10
**KO 3H / KO UNTR**	90	83|7	32	27|5
**KO 6H / KO UNTR**	80	78|2	26	26|0
**KO 12H / KO UNTR**	87	81|6	23	17|6
**KO UNTR / WT UNTR**	41	1|40	30	0|30
**KO 3H / WT 3H**	34	29|5	20	17|3
**KO 6H / WT 6H**	19	9|10	11	5|6
**KO 12H / WT 12H**	31	14|17	20	6|14
